# Atrial septal defect closure as second-line therapy in refractory heart failure: a successful case report in a 6-year-old child with phosphoglucomutase 1 deficiency (PGM1-CDG)

**DOI:** 10.1093/ehjcr/ytaf583

**Published:** 2025-11-06

**Authors:** Sofiane Mimoun, Alban-Elouen Baruteau, Bruno Lefort, Céline Grunenwald, Werner Oscar

**Affiliations:** Nantes Université, CHU Nantes, Department of Pediatric Cardiology and Pediatric Cardiac Surgery, FHU PRECICARE, 7 quai Moncousu, 44093 Nantes cedex 01, Nantes F-44000, France; Nantes Université, CHU Nantes, Department of Pediatric Cardiology and Pediatric Cardiac Surgery, FHU PRECICARE, 7 quai Moncousu, 44093 Nantes cedex 01, Nantes F-44000, France; Nantes Université, CHU Nantes, INSERM, Department of Pediatric Cardiology and Pediatric Cardiac Surgery, CIC FEA 1413, 7 quai Moncousu, 44093 Nantes cedex 01, Nantes F-44000, France; Nantes Université, CHU Nantes, CNRS, INSERM, L’institut du Thorax, 8 Quai Moncousu, Nantes 44007, France; Nantes Université, CHU Nantes, INRAE, UMR 1280 PhAN, Place Alexis Ricordeau. 44093 – NANTES Cedex 1, France; CHU Tours, Department of Pediatric Cardiology, Tours University, FHU PRECICARE, 49 Bd Béranger, 37044 Tours, France; INSERM UMR 1069, 10 Bd Tonnellé, 37000 Tours, France; Nantes Université, CHU Nantes, Department of Pediatric Cardiology and Pediatric Cardiac Surgery, FHU PRECICARE, 7 quai Moncousu, 44093 Nantes cedex 01, Nantes F-44000, France; Nantes Université, CHU Nantes, Department of Pediatric Cardiology and Pediatric Cardiac Surgery, FHU PRECICARE, 7 quai Moncousu, 44093 Nantes cedex 01, Nantes F-44000, France; Nantes Université, CHU Nantes, INSERM, Department of Pediatric Cardiology and Pediatric Cardiac Surgery, CIC FEA 1413, 7 quai Moncousu, 44093 Nantes cedex 01, Nantes F-44000, France

**Keywords:** Atrial septal defect, Percutaneous closure, Left ventricular heart failure, Ventricular compliance, Case report

## Abstract

**Background:**

Atrial septal defect (ASD) is a common congenital heart defect that remains mainly asymptomatic in childhood. However, when left ventricular (LV) compliance is impaired, as in myocarditis or cardiomyopathy, ASD may lead to severe heart failure due to increased left-to-right shunting and a reduced systemic cardiac output.

**Case summary:**

We report the case of a 6-year-old boy with CDG-PGM1 and a large ASD who developed acute refractory heart failure, likely triggered by Parvovirus B19 myocarditis. Despite optimal medical therapy, including immunoglobulins, inotropes, and heart failure medication, he remained dependent on pharmacological support. He was deemed ineligible for heart transplantation due to his underlying metabolic condition; therefore an ASD closure was questioned as a last therapeutic option. A balloon occlusion test showed a mild increase in LV end-diastolic pressure, allowing percutaneous closure using a hand-made fenestrated device. The procedure was successful, leading to rapid weaning from inotropes and full clinical recovery. At 6-month follow-up, LV function was normalized, and the patient was asymptomatic.

**Conclusion:**

In selected patients with impaired LV compliance, ASD closure may offer a life-saving therapeutic option. Haemodynamic evaluation with balloon occlusion testing is essential to evaluate closure feasibility and determine the potential need of using a fenestrated device.

Learning points

- Although atrial septal defect (ASD) is generally benign, left ventricular injury can lead to refractory heart failure from increased left-to-right shunting and reduced systemic output.
- ASD closure should be considered in such cases, with balloon occlusion testing being essential to guide the use of a fenestrated device and prevent post-closure haemodynamic complications.
- CDG-PGM1 syndrome is a rare autosomal recessive disorder that manifests early with multisystemic lesions, notably congenital heart defects and dilated cardiomyopathy, which largely determine prognosis.

## Introduction

Atrial septal defect (ASD) is one of the most common congenital heart diseases and is asymptomatic in childhood in most cases. However, its haemodynamic management may be complicated by acute or chronic left ventricular (LV) injury, which may increase shunt severity by affecting LV compliance.

Congenital disorders of glycosylation (CDG) are a rare metabolic condition characterized by multisystem involvement including neurologic, metabolic, coagulation disorders, musculoskeletal disorders. Phosphoglucomutase-1 deficiency (CDG-PGM1) is a rare subtype often presenting with early cardiac involvement, including congenital heart disease and dilated cardiomyopathy.

We report the case of a 6-year-old boy with CDG-PGM1 and a haemodynamically significant left-to-right (L-R) pre-tricuspid shunt through a large ostium secundum ASD, who gradually developed severe myocardial systolic and diastolic dysfunction and refractory acute heart failure.

## Summary figure

**Figure:**
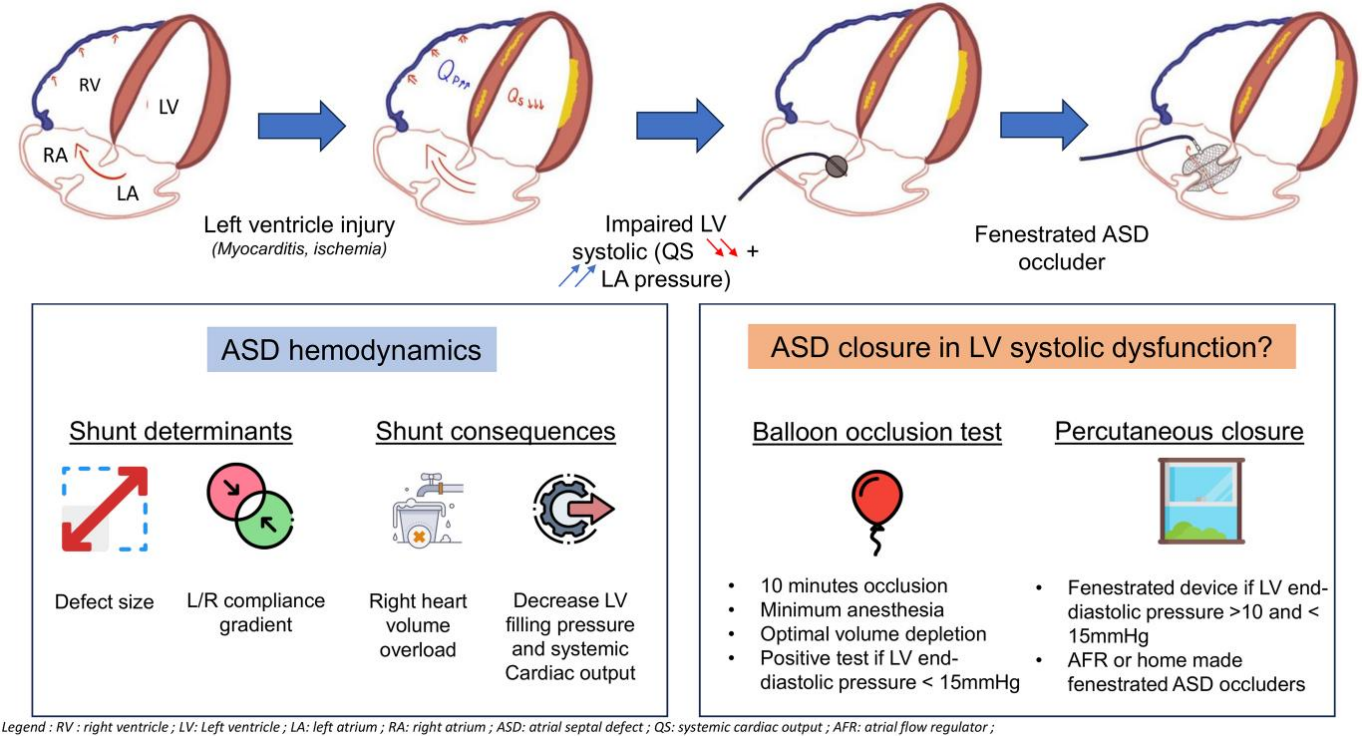


## Clinical presentation

A 6-year-old boy with a known CDG-PGM1 syndrome presented to the emergency department with abdominal pain, fatigue, and hypotonia.

He was followed by a cardiologist for a large secundum ASD without functional limitation. This congenital heart defect was identified during routine cardiovascular screening following the diagnosis of his metabolic disorder. His last echocardiography, six months prior, showed no LV dysfunction or pulmonary hypertension. Extracardiac findings of his CDG syndrome included a Pierre Robin sequence with a previous history of surgical correction, growth retardation, and recurrent hypoglycaemia.

**Figure 1:**
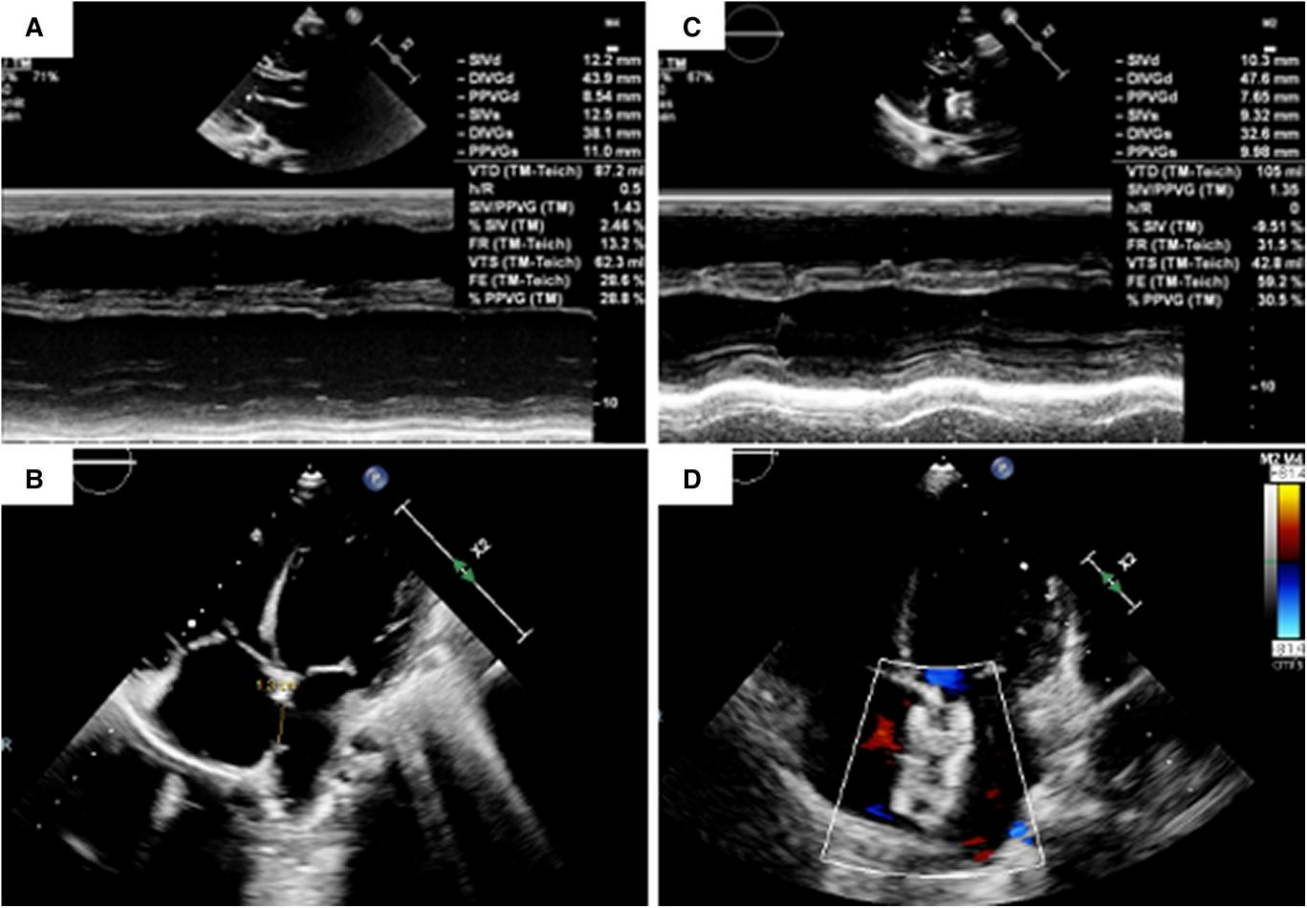
Echocardiographic findings and procedural outcomes: (*A–B*) Initial transthoracic echocardiography showing (*A*) global left ventricular dysfunction with a 25% ejection fraction by Teicholz method, and (*B*) a large, unrestricted atrial septal defect (ASD) with significant right heart chamber enlargement on a 4 chamber- para-sternal view. (*C–D*) Postprocedural echocardiography showing (*C*) an improved left ventricular ejection fraction (40–45% by Teichholz method); after implantation of a 24-mm Amplatzer Septal Occluder with a hand-made 4-mm fenestration (*D*).

Initial clinical evaluation suspected Clostridium difficile colitis, ruled out on abdominal computed tomography, which revealed hepatic congestion and cardiomegaly, suggestive of heart failure. Initial electrocardiogram was unremarkable. Due to rapid clinical deterioration, the patient was referred to the intensive care unit of our paediatric heart transplant centre. In addition to the previously described large and haemodynamically significant ASD, the initial transthoracic echocardiography (TTE) revealed a dilated LV with reduced systolic function, with an ejection fraction of 25% (*Figure 1A* and *B*). Ostia of coronary arteries were normally positioned; cardiac MRI showed diffuse myocardial oedema and LV late gadolinium enhancement suggesting myocarditis. Positive Parvovirus B19 blood serology test supported the hypothesis of acute myocarditis. Management included immunoglobulin therapy, inotropic support (milrinone, adrenaline), and two courses of levosimendan. Given his metabolic condition, D-galactose supplementation was also added to support glycosylation pathways.

As transient improvement in cardiac output was noted, classical heart failure (beta-blockers, diuretics, inhibitors of angiotensin converting enzyme and mineralocorticoid receptor antagonist) and oral anticoagulant therapies were rapidly initiated. However, despite one month of well-conducted heart failure therapy and optimal medical management, the patient remained clinically unstable, with no improvement observed either clinically or on echocardiography. Therefore, a heart transplantation was discussed during a dedicated multidisciplinary team discussion. He was considered at the time ineligible for cardiac transplantation due to his haemodynamic instability and the poor post-transplant prognostic associated to his metabolic condition. Therefore, catheterization was considered to evaluate the feasibility of ASD closure as a last therapeutic option.

**Figure 2:**
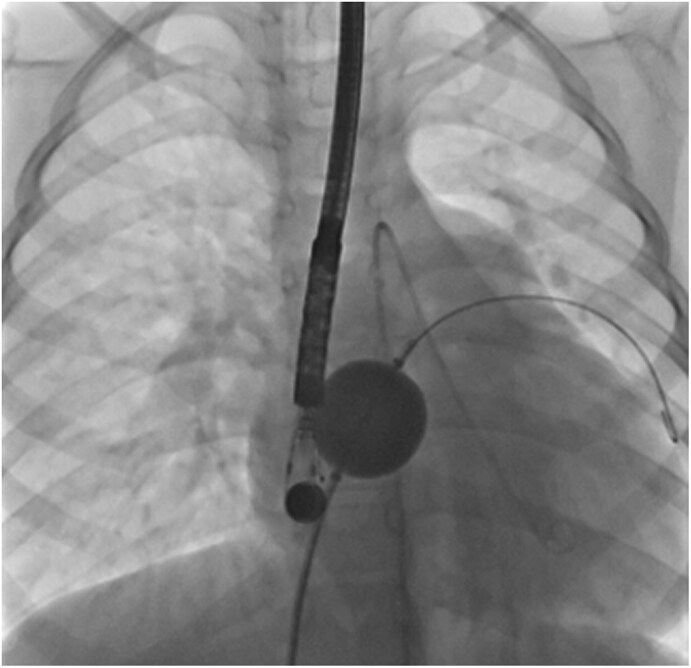
Balloon occlusion test of the atrial septal defect with simultaneous recording of the left ventricular end-diastolic pressure (fluoroscopy, anteroposterior view).

**Figure 3:**
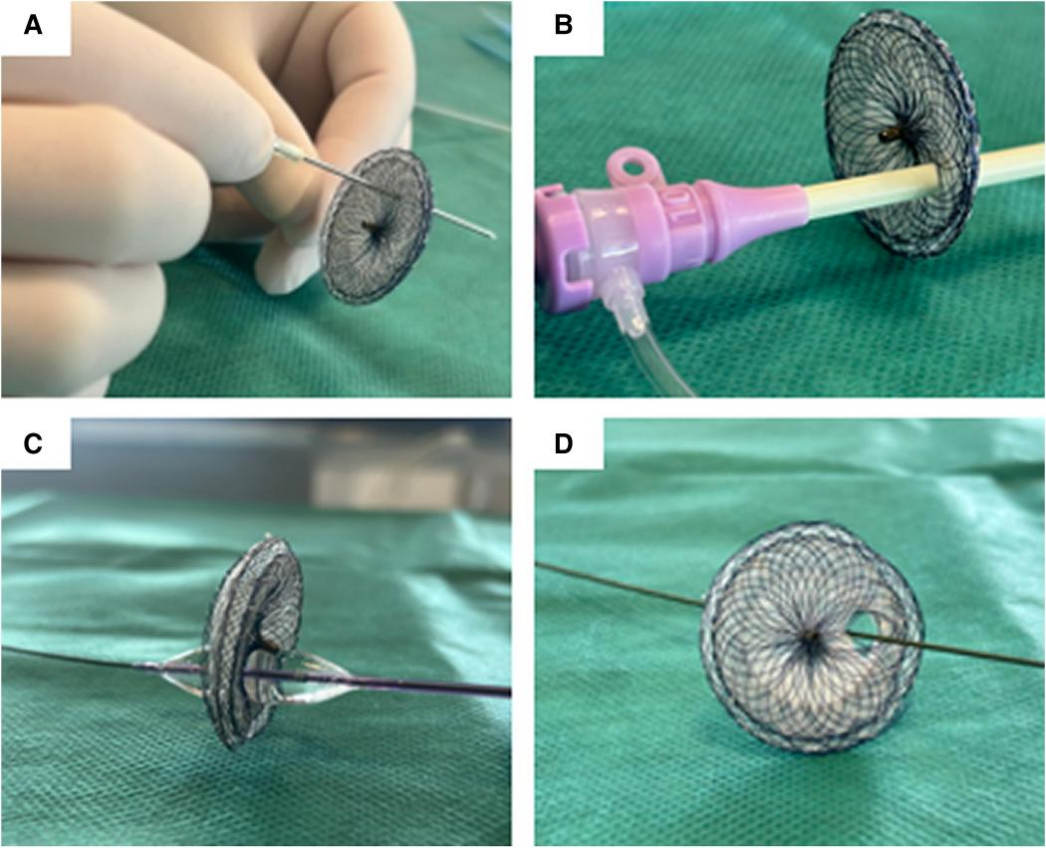
Hand-made fenestration in an ASD Occluder. (*A*) - Initial perforation: A sharp needle is used to puncture the prosthesis, enabling passage of a 0.035 guidewire through the device. (*B*) - Sequential dilation: The hole is then progressively enlarged using 8F to 12-French sheaths. (*C*) - Balloon dilation: A 10 × 20 mm Admiral balloon is inflated to create a controlled 4 mm fenestration, establishing a modified interatrial septal defect. (*D*)– final view of the 4 mm fenestration in the device.

The procedure was performed under general anaesthesia with transoesophageal echocardiographic guidance. Right heart catheterization showed a Qp/Qs ratio of 3.7 and normal pulmonary artery pressures. The ASD measured 15 × 20 mm in diameter in transoesophageal echocardiography without any deficient rim. Baseline LV end-diastolic pressure (LVEDP) was measured at 8 mmHg, and slightly raised to 13 mmHg during ASD balloon occlusion test (*Figure 2*). Consequently, the ASD was deemed closable using a fenestrated device. A 24-mm Amplatzer Septal Occluder with a home-made 4-mm fenestration was successfully implanted (*Figure 3*). At the end of the procedure, the LVEDP was measured at 8 mmHg, and the LV ejection fraction improved immediately at 40–45% by Teichholz method (*Figure 1C* and *D* and Supplementary material). Inotropic support was withdrawn within 24 h. The patient exhibited progressive clinical improvement and was discharged home at postprocedural Day-9. The fenestration closed spontaneously a few weeks after the intervention. At the 6 months outpatient visit, the boy was asymptomatic with normalized cardiac function, and his medical therapies have been reduced to beta-blockers and mineralocorticoid receptor antagonist.

## Discussion

We report the case of a 6-year-old boy with refractory acute heart failure, likely secondary to Parvovirus B19 myocarditis, in the setting of a haemodynamically significant ASD and underlying CDG syndrome.

Physiologically, ASD shunting is determined by the difference between right and left ventricular compliances. Decreased LV compliance (e.g. in myocarditis or myocardial infarction) increases L-R shunting, reduces LV filling, impairs cardiac output and induces RV and pulmonary circulation overload as seen here. It exposes patients to haemodynamic imbalance which can pose major therapeutic challenges. Currently, the guidelines regarding ASD closure recommends to carefully weigh the benefit of eliminating L-R shunt against the potential negative impact of ASD closure on outcome due to an increase in filling pressure.^1^ In this case with severe acute refractory heart failure, we could have considered that the clinical status was not in favour of ASD closure. However, as a heart transplant was contraindicated for this patient, it was decided to perform a balloon occlusion test to assess whether this bailout option was feasible or not. ESC guidelines recommend a 10-minute balloon occlusion test to guide closure decisions (*Figure 2*) to determine whether complete, fenestrated, or no closure is appropriate.^1^ This standardized procedure is considered positive if an increase of at least 10 mmHg in LA or LV pressure is observed which indicates that the shunt should not be closed. To maximize procedural success, optimization of heart failure therapy^2^ and performing this evaluation under minimal or no anaesthesia are required to ensure physiological conditions and allows for precise measurements.^2,3^ It was unfortunately not possible in this 6-year-old boy with limited compliance and unstable haemodynamic state. Here, a modest LVEDP increase (8 to 13 mmHg) was observed and therefore it was decided to use a partially fenestrated ASD occluder to prevent post-closure restrictive physiology. Two types of ASD occluders can be used for such cases, a custom-made or hand-made fenestrated device or the atrial flow regulator (AFR, Occlutech). The choice depends on the defect size as the AFR is not available in large sizes (maximal AFR diameter of 21 mm).^4^ Therefore, we used a home-made fenestrated ASD occluder with several practical steps which are detailed in *Figure 3*.^5,6^ The fenestration is usually expected to close spontaneously within weeks or months.^7^

Finally, recent studies, including Sjöberg *et al.*,^8^ emphasize that early ASD closure improves LV filling and function and may prevent long-term adverse remodelling. This is especially relevant in rare but well-known patients with genetic predispositions to LV cardiomyopathy, with or without ASD-related mutations (*e.g.* NKX2.5, GATA4, or TBX5 (ASD-linked), or sarcomeric mutations (non-ASD-linked)). In such cases, early closure of the ASD should be considered to mitigate the risk of potential complications, if technically feasible, using a percutaneous approach.^9,10^

## Conclusion

ASD closure in the context of heart failure requires thorough invasive haemodynamic assessment to prevent post-closure complications and support ventricular recovery. This case highlights the need for individualized interventional strategies, including the use of a fenestrated device, which can help mitigate reduced LV filling and impaired cardiac output while allowing a residual left-to-right shunt that limits elevations in left atrial pressure.

## Lead author biography


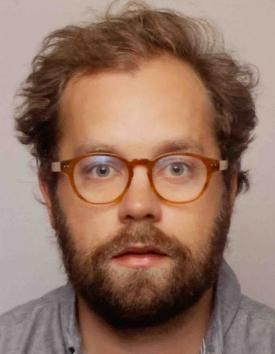


O. WERNER is a paediatric cardiologist specialized in cardiac imaging and is working in a paediatric and congenital cardiac surgery centre in France.

## Supplementary Material

ytaf583_Supplementary_Data

## Data Availability

The datasets presented in the current study are not exposed in the main manuscript but are available from the corresponding author on request.

## Notes

**Consent:** Informed consent was obtained from the parents of the patient.
